# Innovative Bidirectional Isolated High-Power Density On-Board Charge for Vehicle-to-Grid

**DOI:** 10.3390/s22218473

**Published:** 2022-11-03

**Authors:** Roman Hrbac, Libor Hrdina, Vaclav Kolar, Zdenek Slanina, Vojtech Blazek, Tomas Vantuch, Mikołaj Bartłomiejczyk, Stanislav Misak

**Affiliations:** 1 Faculty of Electric Engineering and Computer Science, VSB-Technical University of Ostrava, 17. Listopadu 2172/15, 708 00 Ostrava, Czech Republic; 2 ENET Centre, VSB-Technical University of Ostrava, 17. Listopadu 2172/15, 708 00 Ostrava, Czech Republic; 3 Faculty of Electrical and Control Engineering, Gdansk University of Technology, Narutowicza st. 11/12, 80-233 Gdansk, Poland

**Keywords:** automotive components, AC-DC power converters, automotive electronics, battery chargers, bidirectional power flow, energy conversion, power control, power semiconductor devices, smart grids

## Abstract

This paper deals with developing and implementing a bidirectional galvanically isolated on-board charger of a high-power density. The power density of the new charger was 4 kW/kg and 2.46 kW/dm^3^, and the maximum efficiency was 96.4% at 3.4 kW. Due to the requirement to achieve a high-power density, a single-stage inverter topology was used. Regarding switching losses, due to the topology of the circuit with so-called hard switching, the switching frequency was set to 150 kHz. A laboratory prototype was built to verify the properties and operating principles of the described charger topology. The on-board charger has been tested in a microgrid test platform. Due to the parasitic properties of the transformer and other electronic components, overvoltage with subsequent oscillations occurred on the primary side of the transformer and damped resonance on its secondary side. These parasitic properties caused interference and especially voltage stress on the semiconductor elements. These undesirable phenomena have been eliminated by adding an active element to the charger topology and a new transistor control strategy. This new switching control strategy of transistors has been patented.

## 1. Introduction

Electromobility has become a growing global phenomenon. Not only it has the potential to contribute to the reduction of CO_2_ emissions and improve the environment, especially in large urban agglomerations [1], but it may also provide a solution to the problem of limited fossil fuel reserves. The advances in electromobility concern both passenger cars as well as public transport (electric buses and trolleybuses with battery backup). However, these developments are also accompanied by problematic power engineering and operational character aspects. For the smooth incorporation of electric vehicles, it is necessary to ensure that there is enough electricity and sufficient transmission capacity in the power grid to charge electric vehicles (EVs) when needed or to optimise the charging mode of a significant proportion of EVs so that the electricity sources and distribution grids are able to cover the demand [2].

The battery charging time is significantly longer than the refuelling time of a conventional car [3]. Charging times range from tens of minutes to tens of hours, depending on the specific vehicle type, the battery itself and the charging station [4]. Fast charging is limited, among other things, by the need for high power consumption from the grid and the capacity of the distribution grid at a particular place and time. This problem is solved, for example, by including a battery in the charging station to cover significant power peaks to ensure the fast charging of an EV [5]. The battery in the charger increases its cost, and the temporary energy storage in it reduces the whole system’s efficiency. Currently, several proposed charging-in-motion concepts are associated with several technical and operational problems and have not been widely applied commercially [6].

Currently, the electricity sector is developing the so-called smart grids, enabling a more intense and efficient use of energy sources and distribution grids and better control of their capacity. The implementation of smart grids is currently revolutionizing the distribution and storage of electricity and affecting its generation and consumption [7,8,9].

At the same time, there is a growing importance of renewable sources of electricity, whose performance is in many cases uneven, depending on the actual wind or sunshine, which cannot be influenced, only predicted to a limited extent [10]. Therefore, the need for electricity storage will also increase, which is estimated to triple by 2030 compared to the current situation [11]. One option for short-term electricity storage is to use the battery pack of an electric vehicle connected to the grid via a bidirectional charging station [11,12,13,14]. Such a bi-directional charger can also be designed as a power conditioner [15].

Using EV batteries to support the grid is beneficial only if large EV population is involved [16] and intelligent charging scheduling is also useful [17], which is easier when dealing with a large number of single-owner vehicles. It is therefore desirable to include, for example, huge fleets of public transport EV, such as electric buses, or car-sharing fleets. It was also established that drivers are more open to adopting e-mobility after becoming familiar with the concept through car-sharing. At the same time, easy charging availability is an important factor for EV use, even if it is shared [18,19] The bi-directional on-board charger can find a use in all these applications.

The currently used chargers can be classified according to several criteria. Practically all used chargers must be designed and controlled to respect the limits of current harmonic distortion and power factor [5,20]. Besides the fact that galvanically isolated chargers provide a higher level of safety, their disadvantage is that the transformer increases its weight, size and cost. Moreover, the transformer decreases the overall efficiency of the device. For example, in a galvanically isolated charger operating at 200 kHz, transformer losses are 20% of the total losses [21]. From the point of view of losses, it would be more advantageous to operate the transformer and switch elements at low frequency [22,23].

Many circuit designs allow for a bidirectional power flow with harmonic grid current. One of the simplest is called “totem pole PFC” [24]. It does not contain a transformer, with all consequent advantages and disadvantages (lower power losses, weight, cost, lower level of safety). Furthermore, the battery voltage must always be higher than the main voltage amplitude in both power flow directions. Therefore, it is not a suitable solution for our purpose. 

Series connection of a rectifier with DC/DC converter creates a charger with the so-called two-stage energy transfer [25]. Using this topology in the charging mode, the active front-end rectifier with the sinusoidal current consumption rectifies the input AC voltage. The resulting DC link voltage is converted via an isolated DC/DC converter with an insulating high-frequency (HF) transformer. All the high-power switches need to work with high switching frequency, which causes switching losses proportional to the transmitted power and control technique. 

To reduce switching losses, different topologies were proposed utilizing a zero-voltage switching [26] or resonant effects, for example [27]. Other strategies to reduce power losses of the charger are to reduce the number of energy conversions, reduce the number of HF power switches and passive components that cause losses and increase the weight, dimensions and also price of the device. 

The number of energy conversions is reduced in topologies based on the principle of the matrix converter; this convertor is called single stage [25,28]. This converter supports direct conversion of AC voltage to AC voltage with different parameters. Such energy is subsequently transformed and rectified. This removes the need for DC bus intermediate stage. Input and output bridges work synchronously on the same frequency. All used power switches are switched with high-frequency, and their parameters need to reflect it. HF transistors are characterised with higher RDS in on-state and the higher forward voltage drop across the body diode. 

It is possible to reduce the number of HF switches by optimizing the circuit topology but keeping the single stage energy conversion. The mentioned topologies and their relationship are given in [29]. 

The resulting circuit and its operation are very well described, for example, in [30]. A similar principle was also proposed for a high-power railway application in [31,32].

**Figure 1:**
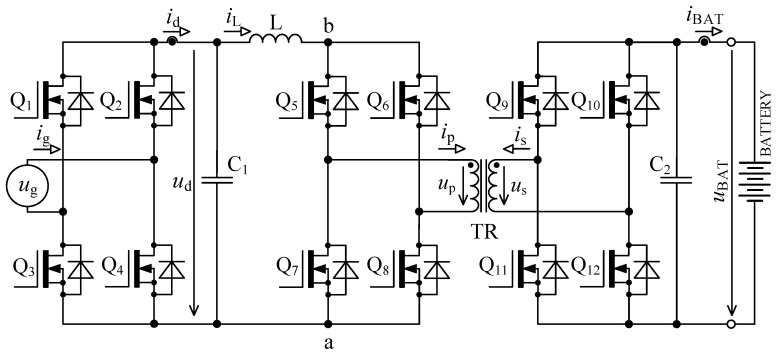
Bidirectional single-stage charger topology [33,39].

The circuit in Figure 1 is based on the topology given in [32,33]. Inductor L is used for energy accumulation in order to be able to transfer the energy from the primary to the secondary side under different magnitudes of *u*_d_. 

The selected topology provides galvanic isolation between the mains and battery sides of the charger’s circuit. Furthermore, it will comply with the following requirements: high-power density, lower number of components, absence of bulky and unreliable electrolytic capacitors, smaller size and lower weight, higher reliability, lower circuit complexity, good dynamic performance, lower harmonic content, high efficiency and lower price in comparison with other typically used topologies. 

Other concepts of bidirectional converters to be utilised in bidirectional chargers can be found in [34]. 

The contribution of this work can be summarised as follows:

- A description of the implementation of the first prototype and its problems related to the circuit topology and parasitic properties of the elements encountered and their solutions not reported in the literature;
- Describe the elimination of voltage spikes using passive disconnectors (RCDs) proved inappropriate due to the significant power losses;
- The introduction of an innovative approach that eliminates voltage spikes based on the use of an applied active clamp in this situation;
- Proposed and described a switching control method that eliminates unwanted HF oscillations on both the primary and secondary sides of the transformer in the on-board charger;
- Description of the development, implementation and testing of a bidirectional charger with galvanic isolation, whose parameters were 4 kW/kg and 2.46 kW/dm^3^ and the maximum power of the charger is 7.2 kW, which uses a switching frequency of 150 kHz.

For comparison, comparable power converters reported in the literature achieve power density of 0.65 kW/dm^3^ [35], 1.875 kW/dm^3^ [36] (gravimetric densities are not specified), 2 kW/dm^3^ with 1.2 kW/kg [37] and 2.44 kW/dm^3^ (gravimetric density is not specified) [38].

This paper is organised as follows. Section 2 deals with the characteristics of the first charger prototype. The relationships for the design of the storage choke are also derived. Section 3 focuses on solving the overvoltage and oscillation problems in the prototype. Relations for calculating the value of the surge eliminating capacitor are given, including the timing of its switching. The simulations were used to check the correctness of the proposed solution set-up. The logic signal waveforms for charging and discharging modes are shown. Section 4 describes the second prototype of the bidirectional charger, including the surge and oscillation suppression circuit. Section 5 described the platform where the on-board charge was laboratory tested. Finally, Section 6 describes the measurements on the second prototype, including the measurement results.

## 2. Verification of Selected Topology of Bidirectional Charger 

When we compare the characteristics of the different topologies of inverters suitable for implementing on-board bidirectional chargers for electric vehicles, the best option seems to be the topology, as shown in Figure 1 [33,39]. 

### 2.1. Calculation of Accumulating Inductor Inductance 

**Figure 2:**
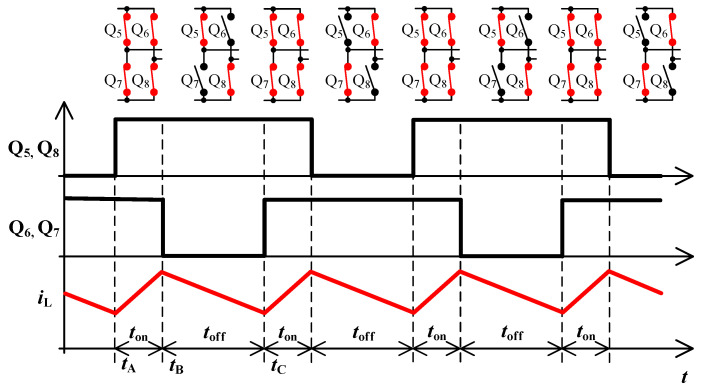
Principle of selected topology from Figure 1 in charging mode. Top-down: waveforms of control pulses Q_5_, Q_8_, control pulses Q_6_, Q_7_, inductor current *i*_L_.

To derive inductance of accumulating inductor, Figure 1 and Figure 2 are considered. The primary purpose of the inductor is to accumulate energy in charging mode, where together with bridge Q_5_ to Q_8_ it forms the so-called boost converter. The switching frequency of transistors Q_5_ to Q_8_ is many times higher than mains frequency. For this reason, the voltage *u*_d_ might be considered constant during a single switching period of these transistors. 

In time interval *t*_on_ the energy is accumulated in inductor L. Voltage *u*_d_ is applied to it and inductor current starts to rise. In time *t*_off_, inductor supplies energy into the battery through the transformer and its current starts to decrease. In time *t*_off_ the inductor voltage is equal to the battery voltage multiplied by transformer ratio *k* and reduced by voltage *u*_d_. The inductor shall be designed so that its current ripple is below the limit. The following equations apply:

(1) $$\Delta i_{L}=\frac{1}{L}\overset{t_{B}}{\underset{t_{A}}{\int }}u_{d}dt=\frac{1}{L}\cdot u_{d}\cdot t_{\mathrm{on}}$$

(2) $$\Delta i_{L}=\frac{1}{L}\overset{t_{C}}{\underset{t_{B}}{\int }}\left(u_{\mathrm{BAT}}\cdot k-u_{d}\right)dt=\frac{1}{L}\left(u_{\mathrm{BAT}}\cdot k-u_{d}\right)\cdot t_{\mathrm{off}}$$

where *k* = 1.33 is the transformer ratio.

Figure 2 implies the equation for the inductor current period:

(3) $$T_{L}=t_{\mathrm{on}}+t_{\mathrm{off}}=\frac{1}{2\cdot f}$$

Combining (1), (2) and (3), the equations below are derived:

(4) $$t_{\mathrm{on}}=\frac{u_{\mathrm{BAT}}\cdot k-u_{d}}{u_{d}}\cdot t_{\mathrm{off}}$$

(5) $$t_{\mathrm{on}}=T_{L}\cdot \frac{u_{\mathrm{BAT}}\cdot k-u_{d}}{u_{d}}-t_{\mathrm{on}}\cdot \frac{u_{\mathrm{BAT}}\cdot k-u_{d}}{u_{d}}$$

(6) $$t_{\mathrm{on}}=T_{L}\cdot \frac{u_{\mathrm{BAT}}\cdot k-u_{d}}{u_{\mathrm{BAT}}\cdot k}$$

Combining (1), (3) and (6), the equation below is derived:

(7) $$\Delta i_{L}=u_{d}\cdot \frac{u_{\mathrm{BAT}}\cdot k-u_{d}}{L\cdot 2\cdot f\cdot u_{\mathrm{BAT}}\cdot k}$$

Equation (7) can be used to derive the equation for inductance:

(8) $$L=u_{d}\cdot \frac{u_{\mathrm{BAT}}\cdot k-u_{d}}{\Delta i_{L}\cdot 2\cdot f\cdot u_{\mathrm{BAT}}\cdot k}$$

The switching frequency of the charger is 150 kHz. For a ripple current of 10 A, a choke inductance of 45 μH was calculated according to Equation (8). Due to the fact that at this inductance value, the inductor’s core was oversaturated, a value of 25 μH was finally chosen experimentally. For this inductance, its ripple current reaches up to 20 A.

### 2.2. Experimental Results Measured on the First Prototype of Bidirectional Charger

To verify the selected topology of the charger and its proposed control principles, a laboratory prototype was built.

The prototype was assembled from three separate printed circuit boards: control, driver and power. The control board contains:

- Microcontroller EFM8LB12F64E-QFP32 from Silicon Labs.
- Supporting circuits for logic of control pulses and elimination of various switching failure modes.
- Regulated power supplies with various output voltages.

The power board contains:

- Power transistors, capacitors, planar transformer and inductor. With respect to the required dimensions and overall weight of the device cores, E64/10/50-3F36 and PLT64/50/5-3F36 were used. A detailed list of the used components is given in Section 4.

**Figure 3:**
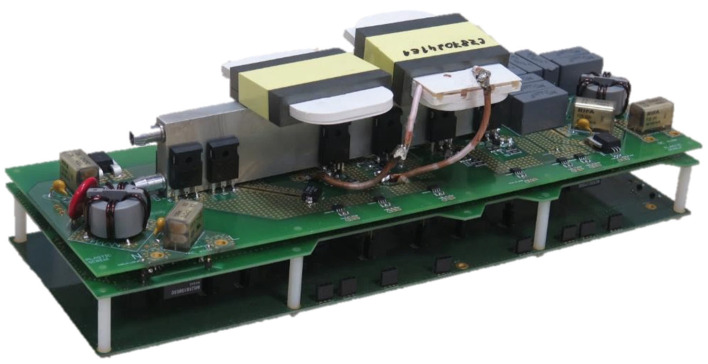
A photograph of the first prototype of the bidirectional isolated on-board charger.

Figure 3 shows the first prototype of the single stage bi-directional isolated on-board charger with its three PCBs in sandwich-like assembly.

**Figure 4:**
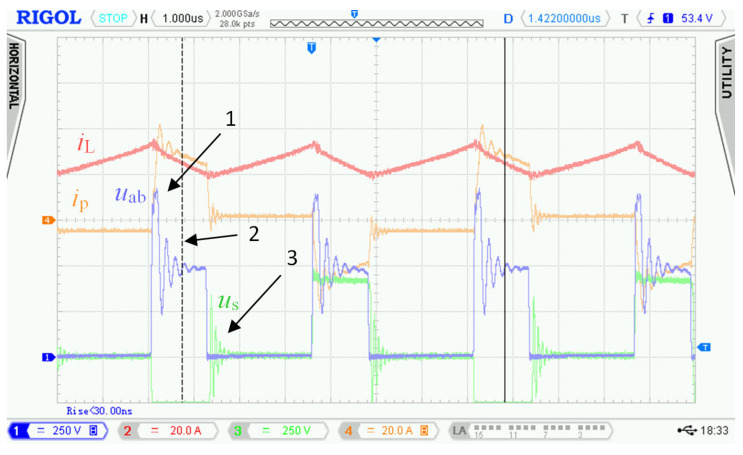
Waveforms measured with the first prototype of bidirectional charger in charging mode. Measured at: *u*_d_ = 175.5 V, *i*_d_ = 25.8 A, *u*_BAT_ = 397 V*, i*_BAT_ = 9.95 A, *P*_BAT_ = 3950 W and inductor inductance *L* = 25 μH.

The waveforms in Figure 4 show transient phenomena in the circuit during two switching periods. After the switch-off of the first transistor pair Q_5_, Q_8_, the voltage starts to rise (Figure 4, point 1) on the primary side of the transformer and at the same time also, its current starts to rise. Transformer TR (see Figure 1) is not able to immediately pass the inductor L current due to its leakage inductance. This causes overvoltage followed by oscillations (Figure 4, point 2). The energy transfer through the transformer ends when all transistors Q_5_ to Q_8_ have switched on again.

After the primary voltage of the transformer decreases to zero volts, a decrease in its secondary side voltage follows. A steep decrease in the secondary voltage induces dumped oscillations due to parasitic circuit properties (Figure 4, point 3). Overvoltage and oscillations are not only the source of EMI noise but cause voltage to overstress to power transistors, which could lead to their destruction. For this reason, we had to find a solution to eliminate the oscillations and reduce the overvoltage.

## 3. Selected Topology Troubleshooting

**Figure 5:**
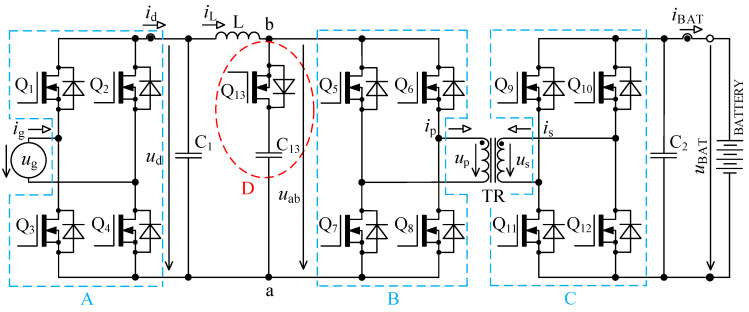
Topology of bidirectional charger extended with an active element “D” consisting of a transistor Q_13_ and capacitor C_13_.

In Figure 5 there is a principal scheme of the bidirectional charger, to which relate all the following descriptions of logic signals of transistor Q_1_ to Q_13_ and all experimental measurements.

As was already described in Section 2, using the bidirectional topology charger from Figure 1 and driving transistors according to Figure 2, there are two major issues:

Overshoot of voltage *u*_p_ (Figure 4, point 1) and oscillations (Figure 4, point 2) on the primary winding of transformer TR caused by its parasitic properties.

Damped resonance of parasitic circuit elements on the secondary side of the transformer (Figure 4, point 3) caused by steep decrease in primary voltage *u*_p_.

The first problem with voltage *u*_p_ overshoots was successfully solved by implementing an active circuit element marked “D” in Figure 5 and its exact timing. This active element consists of series connection of transistor Q_13_ with capacitor C_13_. Transistor Q_13_ switches in the capacitor C_13_ at exactly defined time period.

The second problem with damped resonance of parasitic circuit elements on the secondary side of the transformer was eliminated by a different control technique of transistors Q_9_ to Q_13_ (different to the control shown in Figure 2).

### 3.1. Root Cause Description of Overvoltage on the Primary Side of Transformer and Solution

When the transistor pair Q_5_, Q_8_ or Q_6_, Q_8_ is switched off, inductor L acts as a source of constant current. In the ideal case, this current would start to flow through the primary winding of transformer, but its leakage inductance prevents that. In case the active element “D” (see Figure 5) is missing, the current will not have a path to flow, and voltage will rise between nodes a and b. This would lead to severe overvoltage. 

**Figure 6:**
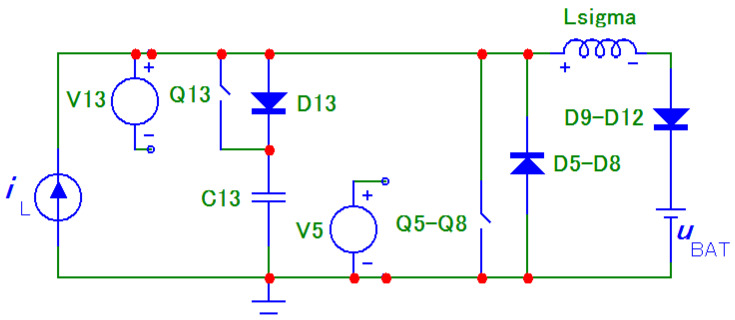
Equivalent scheme for simulation of transient effect with active control of capacitor C_13_.

The moment of switching off the transistor pair Q_5_, Q_8_ or Q_6_, Q_7_ could be described based on Figure 6, which represents circuit schemes in simulation software Microcap, which was used to verify the circuit behaviour.

At the moment of switching off transistor pair Q_5_, Q_8_ (Q_6_, Q_7_), the transistor Q_13_ is also switched off. See Figure 2, time *t*_0_ (*t*_0_ + ½*T*). The inductor current *i*_L_ flows immediately through internal body diode D13. For the purpose of simplification, its magnitude could be considered constant over the duration of transient. At the same time, the current through leakage inductance *L*_sigma_ starts to rise. In time *t*_1_ (*t*_1_ + ½*T*) transistor Q_13_ turns on.

Capacitor C_13_ and leakage inductance *L*_sigma_ together form a resonant circuit. Its resonant frequency is given by (9).

(9) $$f_{r}=\frac{1}{2\pi }\sqrt{\frac{1}{C_{13}\cdot L_{\mathrm{sigma}}}}$$

During the transient, the energy accumulated in inductance *L*_sigma_ is:

(10) $$E=\frac{1}{2}L_{\mathrm{sigma}}\cdot i_{L}^{2}$$

The same amount of energy accumulates in the capacitor C_13_, which is based on the general knowledge about the behaviour of resonant LC circuits:

(11) $$E=\frac{1}{2}C_{13}\cdot \Delta U_{C13}^{2}$$

Let us assume that in *t*_0_ the capacitor C_13_ is charged to voltage *k*·*u*_BAT_ from the previous period, where *k* is transformer ratio. The following equation is obtained using (10) and (11):

(12) $$U_{C13\max}=k.u_{\mathrm{BAT}}+\sqrt{\frac{L_{\mathrm{sigma}}}{C_{13}}}\cdot i_{L}$$

Neglecting the voltage drop across the body diode D_13_ and transistor Q_13_ (see Figure 5), Equation *u*_C13max_ = *u*_abmax_ applies. If the maximum *u*_abmax_ voltage is specified (for example based on breakdown voltage of semiconductors), required C_13_ capacity can be calculated as:

(13) $$C_{13}=L\cdot {\left(\frac{i_{L}}{U_{C13\max}-k.u_{\mathrm{BAT}}}\right)}^{2}$$

For the capacitor C_13_ to remain charged to a voltage of magnitude *k*·*u*_BAT_ during the end of the transient effect, exactly one-half period of resonant effect needs to occur. In time *t*_0,_ charging of capacitor occurs through diode D_13_. Discharging occurs through transistor Q_13_, which needs to be turned off at specific time. The total time of current conduction through the capacitor must be half of the period of the resonant frequency.

(14) $$t_{Q13}=\pi \sqrt{C_{13}\cdot L_{\mathrm{sigma}}}$$

Time *t*_Q13_ cannot be longer than the switch-off duration of one of the transistor pairs from bridge “B”, further reduced by safety margin time *t*_delay_, which corresponds to (½*T* − *t*_ov_ − *t*_delay_). In that case, the time *t*_Q13_ is shorter compared to (14):

(15) $$t_{Q13}=\frac{1}{2}T-t_{\mathrm{ov}}-t_{\mathrm{delay}}$$

For time interval when *t*_ov_ < (½*T* − *t*_delay_), (14) applies.

For time interval when *t*_ov_ ≥ (½*T* − *t*_delay_), (15) applies.

It is desirable to design the capacitance of C_13_ so that there is no voltage breakdown of semiconductors during the maximum instantaneous current *i*_L_ magnitude and during maximum battery voltage.

It was decided that the highest voltage amplitude *u*_abmax_ is in the range 650 V to 700 V. Review of parameters for which C_13_ was designed:

Leakage inductance *L*_sigma_ of used transformer is 1 μH, maximum battery voltage is 420 V, transformer ratio is 1.33, maximum inductor current amplitude *i*_L_ is below 50 A. 

For the given voltage *u*_abmax_ amplitude, the C_13_ capacitance was calculated according to (13) in the range from 125 nF to 308 nF. Capacity 270 nF was chosen, for which the duration was calculated as *t*_Q13_ = 1632 ns. The calculations were verified by simulations, see Figure 6.

Inductor L is replaced with a constant current source *i*_L_ in the scheme above. Its value can either be set as a constant value or as a periodic triangular waveform. Transistors were replaced by switches controlled by voltage sources (V5 to V13) with configurable switching diagram. A switch marked as Q_5_ to Q_8_ substitutes transistors of bridge “B”, and diode D5 to D8 represents their body diodes.

The purpose of the first simulation was to verify the amplitude voltage spike during maximum current *i*_L_ = 50 A and maximum battery voltage *u*_BAT_ = 420 V. Simulation is performed for 150 kHz inverter switching frequency therefore 300 kHz frequency of inductor current, *L*_sigma_ = 1 μH, *C*_13_ = 270 nF. Current source *i*_L_ supplies constant current with magnitude 50 A. Time *t*_Q13_ was configured to be 1632 ns.

**Figure 7:**
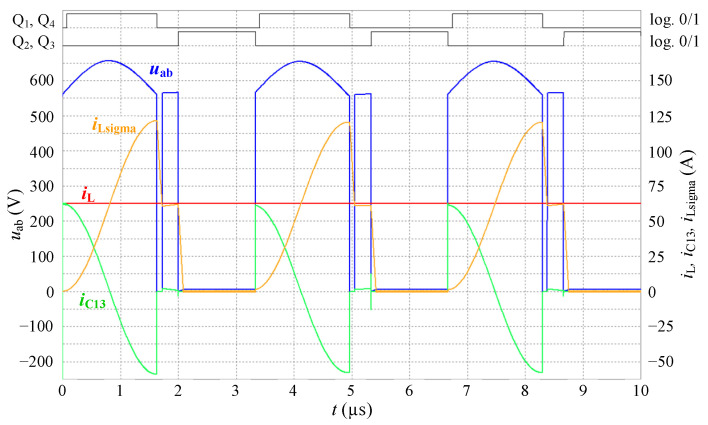
Waveforms of voltages and currents during transient effect as simulated in Microcap software for *u*_BAT_ = 420 V, *i*_L_ = 50 A, *t*_Q13_ = 1632 ns, *t*_ov_ = 1327 ns, *t*_delay_ = 70 ns.

Simulated waveforms of voltages and currents are shown in Figure 7. Substituting the values into (13) the resultant voltage amplitude of *u*_abmax_ = 656.2 V. From the simulation results, the value of 654.4 V was obtained. This is almost a full match to the calculated value.

Another simulation was performed in order to confirm the correctness of (9) to (15) and the overall functionality of the proposed solution. Specific values of voltages and currents, which were measured during experimental measurements with bidirectional charger, were simulated.

The following parameters are considered:

Periodic waveform *i*_L_ of triangular shape with values *i*_Lmin_ = 24 A, *i*_Lmax_ = 43 A, *u*_BAT_ = 371 V. The *t*_ov_ overlap time was 1833 ns. The switch-off time interval of the transistor pair of bridge “B” was 1430 ns, for this reason, according to (15), the time *t*_Q13_ was shortened to 1430 ns. 

**Figure 8:**
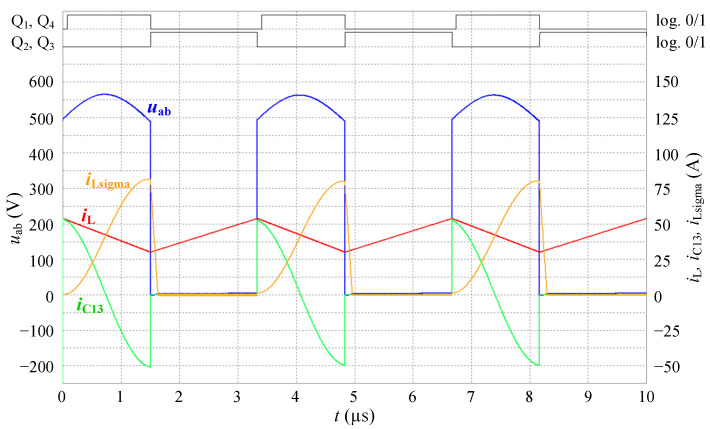
Waveforms of voltages and currents of transient effect as simulated in Microcap software for values *u*_BAT_ = 371 V, *i*_Lmin_ = 24 A, *i*_Lmax_ = 43 A, *t*_Q13_ = 1430 ns, *t*_ov_ = 1833 ns, *t*_delay_ = 70 ns.

The simulation result is shown in Figure 8. The experimentally measured values are shown in Section 6. The simulated and experimentally measured waveforms clearly show the agreement. The simulations also verified the correctness of the derived equations.

### 3.2. Principle of the New Strategy of Bidirectional Charger Control in the Charging Mode

In order to achieve higher efficiency, transistors Q_1_ to Q_4_ (bridge “A”) are actively driven with mains frequency 50 Hz and work as an active rectifier. Transistors Q_1_ to Q_4_ always switch in pairs Q_1_, Q_4_ and Q_2_, Q_3_. Correct switching of those transistor pairs is dependent on the polarity of instantaneous mains voltage and is activated after zero crossing. 

Switching period *T* is in the order of units of μs, which relates to switching frequency in order of hundreds of kHz. Switching period for transistor Q_13_ is two times shorter (twice the frequency). Transistors Q_5_ to Q_8_ (bridge “B”) always switch on all together during energy accumulation into inductor L and during the energy transfer the pair Q_5_, Q_8_, or Q_6_, Q_7_ is switched on. For the following explanations, it is assumed that time *t*_0_ starts a new period when the energy transfer from inductor L through transformer TR (see Figure 5) into the battery occurs. The circuit is in a dynamically stabilised state from the previous period. Energy is accumulated in inductors and capacitors.

**Figure 9:**
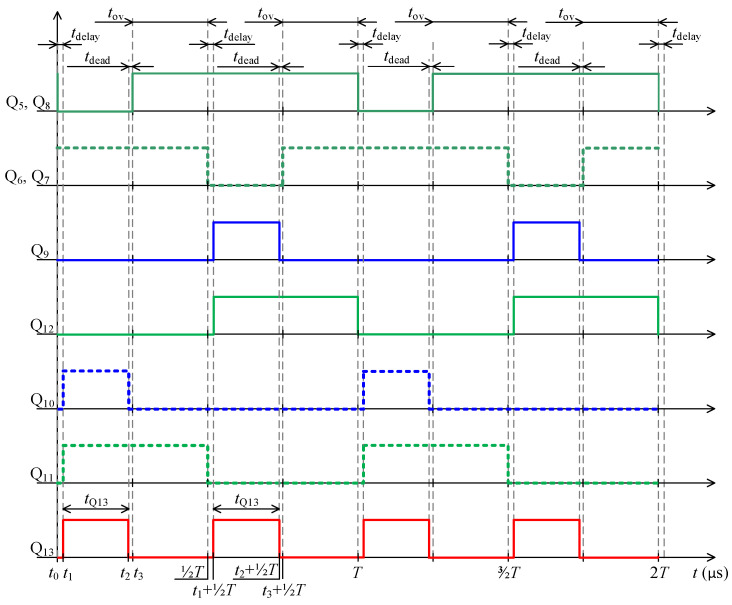
Waveforms of control signals for charging cycle.

**Figure 10:**
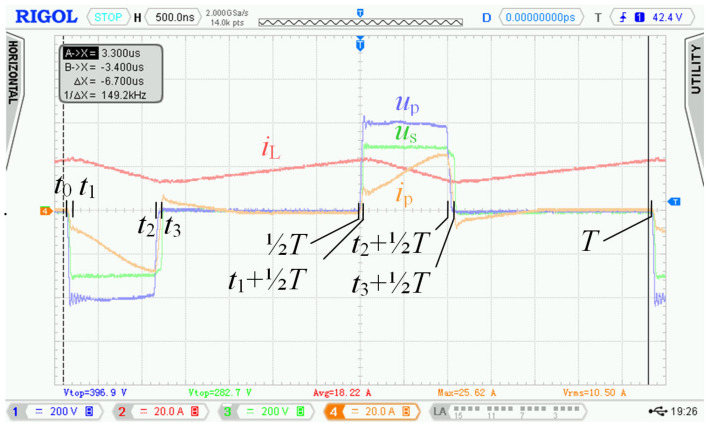
Experimentally acquired waveforms during charging mode; time interval *t*_0_ ≤ *t* ≤ *T*, where *t*_ov_ = ⅜*T*.

Specific switching time intervals for transistors Q_5_ to Q_13_ for charging and discharging mode are described below. Logic diagrams of these transistors switching are shown in Figure 9 for better clarity. Figure 10 shows experimentally acquired waveforms of voltages and current in specific time intervals. All these waveforms are relevant only for charging mode. Important parameter in charging mode is overlap duration *t*_ov_. During this time, all four transistors Q_5_ to Q_8_ are switched on and energy is being accumulated into inductor L. Duration marked as *t*_delay_ is achieved with shifting of microcontroller timers.

This duration needs to be applied when transistors Q_9_ to Q_12_ are actively driven due to a delay in energy transfer from the primary to the secondary winding of the transformer. Duration of *t*_delay_ needs to be long enough to guarantee that voltage generated on the secondary winding is higher than the battery voltage *u*_BAT_. Without *t*_delay_ the current would flow from the battery through transistor pair Q_9_, Q_12_ or Q_10_, Q_11_ first, and later it would be overdriven by current generated in the transformer’s secondary winding. Time interval marked as *t*_dead_ guarantees reliable turn off of all transistors connected in common part of the circuit. It shall never occur that transistors Q_9_ and Q_11_ or Q_10_ and Q_12_ switch on at the same time.

#### 3.2.1. Time Interval *t*_0_ ≤ *t* < *t*_1_

**Figure 11:**
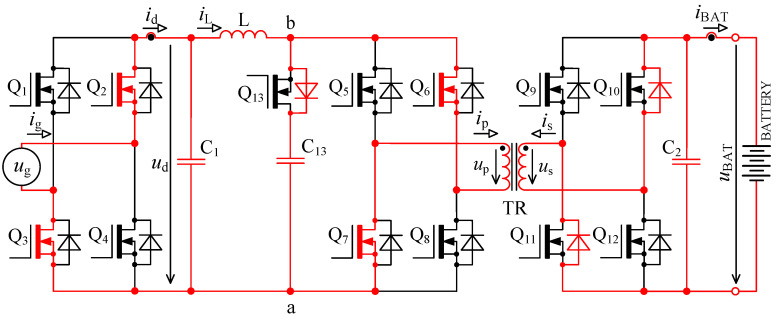
Charging mode–time interval *t*_0_ *≤ t < t*_1_.

In time *t*_0_ transistor pair Q_5_, Q_8_ was turned off while transistors Q_6_, Q_7_ remained turned on from the previous period. Transistor Q_13_ is turned off. Additionally, transistor Q_12_ was turned off. All the transistors in bridge “C” are turned off. The explanation refers to Figure 9, Figure 10 and Figure 11.

Current *i*_L_ flows through inductor L while the primary winding current *i*_p_ of transformer TR is zero. In an ideal case, the inductor L current should immediately flow into the primary winding of transformer TR, but this is not possible due to its leakage inductance. Inductor current *i*_L_ continues through the body diode of transistor Q_13_ into capacitor C_13_, which charges *u*_p_ and accumulates energy. In case of C_13_ absence, the voltage between a and b nodes would rise to dangerous overvoltage, leading to transistor destruction. The active element, composed of a series connection of transistor Q_13_ and capacitor C_13_, prevents that. Current *i*_s_, which is induced by current increase *i*_p_, is flowing back through the body diodes of transistors Q_10_ and Q_11_. In this time the, bridge “C” works as a passive diode bridge rectifier.

#### 3.2.2. Time Interval *t*_1_ ≤ *t* < *t*_2_

**Figure 12:**
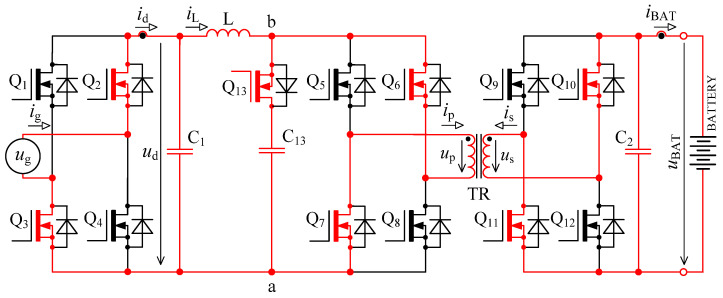
Charging mode–time interval *t*_1_ *≤ t < t*_2_.

The time *t*_1_ (*t*_delay_) is very short, i.e., units of percent of the switching period (150 kHz). In this case, it was 100 ns. The explanation refers to Figure 9, Figure 10 and Figure 12. The primary purpose of this *t*_1_ is to add two safety delays:

- Between switching off transistor pair Q_5_, Q_8_ and switching on Q_3_, while transistors Q_6_, Q_7_ remain switched off. Simultaneous switching on of all transistors in bridge “B” would mean short circuit of capacitor C_13_.
- Guarantees that voltage generated across the secondary winding of the transformer is greater than battery voltage *u*_BAT_.

In time *t*_1_ transistors Q_10_, Q_11_ and Q_13_ turn on. Current *i*_p_ is rising and is now given by the difference of currents *i*_L_ and capacitor current *i*_C13_. After some time, both currents *i*_L_ and *i*_p_ equalise and in this moment direction of current through C_13_ reverses. The energy which was accumulated in C_13_ is now being discharged into transformer TR. Current *i*_p_ is now given by the sum of capacitor *i*_C13_ and inductor *i*_L_ currents. In this time interval, the energy is transferred from the primary to the secondary winding of transformer TR. Transistors Q_10_ and Q_11_ were turned on to reduce losses during the secondary current flow.

#### 3.2.3. Time Interval *t*_2_ ≤ *t* < *t*_3_

**Figure 13:**
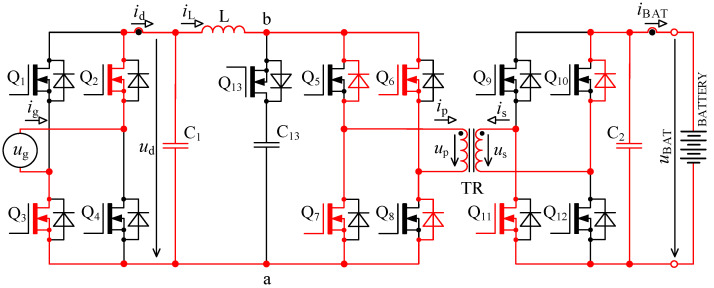
Charging mode–time interval *t*_2_ ≤ *t* < *t*_3_.

In time *t*_2_ transistors Q_10_ and Q_13_ were turned off, while the capacitor C_13_ was disconnected from the bridge “B”. The current *i*_p_ dropped. The leakage inductance of the transformer tries to keep its current magnitude. For this reason, the polarity of *u*_p_ is reversed, but body diodes of transistors Q_5_ and Q_8_ clamp the voltage to approximately zero. The explanation refers to Figure 9, Figure 10 and Figure 13.

Considering that in time *t*_2_ transistor Q_10_ is turned off, the energy accumulated in the leakage inductance of transformer keeps up the current *i*_s_, which flows through Q_10_ body diode.

#### 3.2.4. Time Interval *t*_3_ ≤ *t* < ½*T*

In *t*_3_ instant, the transistors Q_5_ and Q_8_ were turned on, while transistors Q_6_, Q_7_ and Q_11_ remained turned on. Transistors Q_5_, Q_8_ are turned on at zero voltage, which was reached during the previous time interval.

The primary winding of transformer TR is shorted out with transistors Q_5_ to Q_8_, current *i*_p_ decreases to zero. The instantaneous voltage of rectified grid voltage *u*_d_ is now connected to inductor L, its current rises, and energy is being accumulated. The accumulation phase remains till ½*T*. The time during which the energy is being accumulated into the inductor is marked as *t*_ov_.

In order to increase the amount of energy accumulated in inductor L, which is transferred to the secondary winding of transfer, the overlap duration *t*_ov_ needs to be extended while *t*_3_ and *t*_2_ shortened.

**Figure 14:**
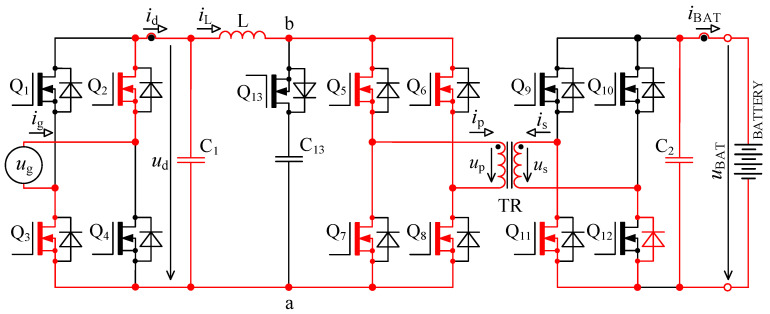
Charging mode–time interval *t*_3_ *≤ t <* ½*T*.

During the accumulation period, the transistor Q_11_ from bridge “C” remains turned on and, together with the body diode of transistor Q_12_ keeps the secondary voltage *u*_s_ close to zero till the moment when no more energy is transferred into the battery. The explanation refers to Figure 9, Figure 10 and Figure 14.

#### 3.2.5. Time Interval ½*T* ≤ *t* < (*t*_1_ + ½*T*)

Time ½*T* is half of the switching period when everything starts to repeat. The main difference is that transistor pairs Q_5_, Q_8_ and Q_6_, Q_7_ switched their roles.

In time ½*T* transistor pair Q_6_, Q_7_ was turned off, while transistors Q_5_, Q_8_ remained turned on from the previous period. Transistor Q_13_ is turned off. Furthermore, transistor Q_11_ was turned off. All the transistors from bridge “C” are now turned off. Waveforms of voltages and currents are analogous to what was described in Section 3.2.1.

#### 3.2.6. Time Interval (*t*_1_ + ½*T*) ≤ *t* < (*t*_2_ + ½*T*)

In time (*t*_1_ + ½*T*), transistors Q_9_, Q_12_ and Q_13_ were turned on. Transistors Q_9_, and Q_12_ were turned on in order to reduce conduction losses during the secondary current flow. Waveforms of voltages and currents are analogous to what was described in Section 3.2.2.

#### 3.2.7. Time Interval (*t*_2_ + ½*T*) ≤ *t* < (*t*_3_ + ½*T*)

In time (*t*_2_ + ½*T*), transistors Q_9_ and Q_13_ were turned off. Transistors Q_5_, Q_8_ and Q_12_ remained turned on. Waveforms of voltages and currents are analogous to what was described in Section 3.2.3.

#### 3.2.8. Time Interval (*t*_3_ + ½*T*) ≤ *t* < *T*

In time (*t*_3_ + ½*T*), transistors Q_6_ and Q_7_ were turned on. Transistors Q_5_, Q_8_ and Q_12_ remained turned on. Waveforms of voltages and currents are analogous to what was described in Section 3.2.4.

### 3.3. Principle of the New Strategy of Bidirectional Charger Control in the Discharging Mode

In discharging mode of a bidirectional charger, transistors Q_1_ to Q_4_ (bridge “A” in Figure 5) and transistors Q_9_ to Q_12_ (bridge “C” in Figure 5) work as inverters. Transistors Q_5_ to Q_8_ (see bridge “B” in Figure 5) work as rectifiers. Transistors Q_9_ to Q_12_ chop the direct voltage of battery *u*_BAT_ into AC voltage, which is later transformed by transformer TR and actively rectified by transistors Q_5_ to Q_8_.

The voltage *u*_d_ itself does not have a constant magnitude, but it is modulated with change of *t*_on_ parameter in such a way that it resembles rectified sine wave. Change of *t*_on_ is realised by switching on the duration of transistor pairs Q_9_, Q_12_ or Q_10_, Q_11_ (inverter “C” in Figure 5), while transistors Q_12_ and Q_11_ are switched on for the whole duration of switching half-period. Only after *t*_delay_ transistor pairs Q_5_, Q_8_ or Q_6_, Q_7_ (inverter “B” in Figure 5) are switched on.

The function of inverter “A” is to toggle the polarity of direct pulsing voltage *u*_d_ so that it reflects the actual polarity in power grid and thus allows the energy to flow from the battery into power grid.

Labelling of transformer windings is kept the same as in charging mode in the Subsection B. Winding of transformer on the battery side is labelled as the secondary winding even though its function in discharging mode is inverted.

Switching period *T* has μs unit, which reflects the switching frequency in the order of hundreds of kHz. The switching frequency of 150 kHz is used in the example below.

**Figure 15:**
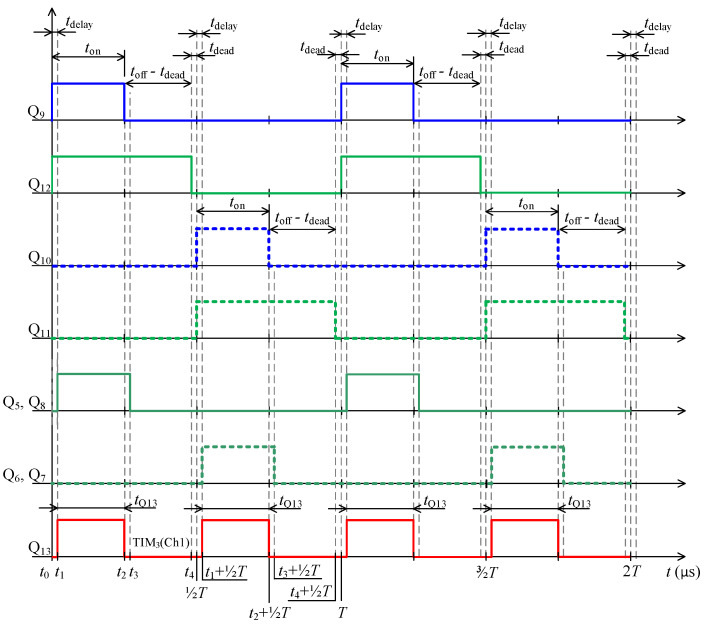
Waveforms of control signals for discharging cycle, *t*_on_ = ¼*T*.

**Figure 16:**
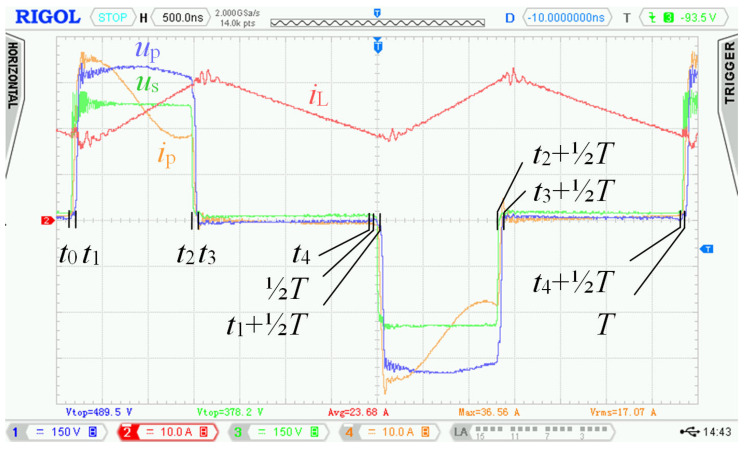
Experimental measurement in discharging mode–time interval *t*_0_ ≤ *t* < *T, part a*.

**Figure 17:**
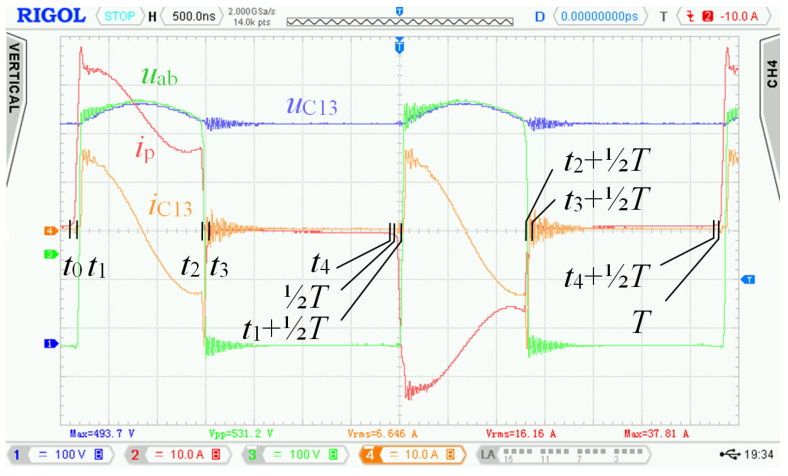
Experimental measurement in discharging mode-time interval *t*_0_ ≤ *t* < *T, part b*.

In the following example, it is assumed that time *t*_0_ is the start of a new period and that the circuit is in a dynamically stabilised state from the previous period, inductors and capacitors have accumulated energy. For clarity, Figure 15 shows the logic switching waveforms of transistors. Figure 16 and Figure 17 show experimentally acquired waveforms of voltages and currents in specific time instants. All those waveforms are relevant only for discharging mode. The main parameter for discharging mode is marked as *t*_on_. During this time one of the transistor pairs Q_9_, Q_12_ or Q_10_, Q_11_ is switched on and energy is transferred via transformer TR and inverter “B” and “A” into power grid. Duration marked as *t*_delay_ for switching of transistor Q_13_ is realised by a phase shift of timer of microcontroller and for transistors Q_5_ to Q_8_ by utilizing *t*_dead_ in the microcontroller.

Voltage spikes are reduced with capacitor C_13_, first passively and later after *t*_delay_ through switched-on transistor Q_13_. In the specific moment, the accumulated energy of C_13_ discharges back into the circuit through switched-on transistor Q_13_.

#### 3.3.1. Time Interval *t*_0_ ≤ *t* < *t*_1_

**Figure 18:**
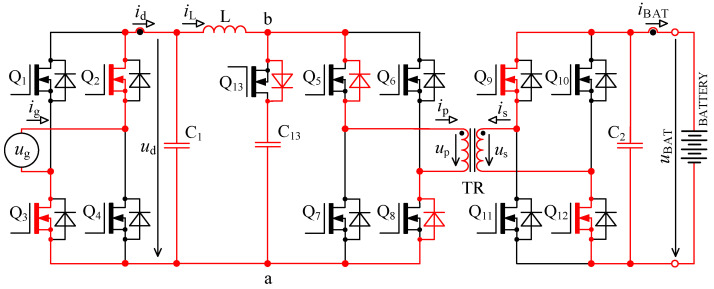
Discharging mode–time interval *t*_0_ *≤ t < t*_1_.

In moment *t*_0,_ transistors Q_9_, Q_12_ are turned on. Transistors Q_5_ to Q_8_, Q_10_, Q_11_ remain turned off. The secondary side of the transformer TR is connected to battery voltage *u*_BAT_. Due to this, the secondary and later also the primary current starts to flow through the transformer TR, which causes voltage *u*_p_ to rise. Current *i*_p_ flows back through the body diodes of transistors Q_5_ and Q_8_. The explanation refers to Figure 15, Figure 16, Figure 17 and Figure 18.

Primary voltage *u*_p_ reaches the magnitude which corresponds to the product of battery voltage and transformer TR transforming ratio. If the voltage u_p_ exceeds the voltage of capacitor C_13_, the current starts to flow through the body diode of transistor Q_13,_ and capacitor C_13_ is charging. At this moment, energy is transferred into power grid and capacitor C_13_.

#### 3.3.2. Time Interval *t*_1_ ≤ *t* < *t*_2_

**Figure 19:**
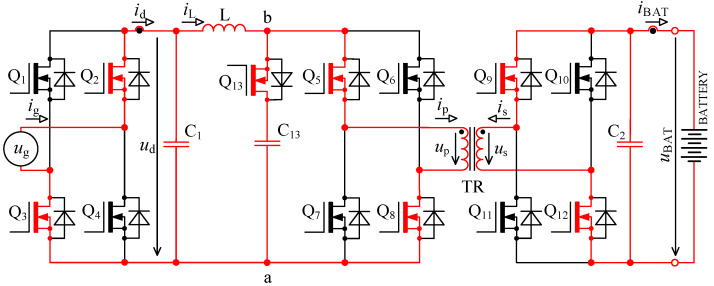
Discharging mode–time interval *t*_1_ ≤ *t* < *t*_2_.

In time instant t_1_ transistors Q_5_, Q_8_, Q_13_ are turned on to reduce conduction losses across their body diodes. The delay of switching on the transistors *t*_delay_ is implemented due to the leakage inductance of transformer TR. Voltage *u*_p_ is greater than instantaneous voltage *u*_d_, and inductor current *i*_L_ is rising. Capacitor C_13_ is charging, and its current decreases. In a moment when the currents *i*_p_ and *i*_L_ have the same magnitude, the current’s direction through C_13_ reverses and its accumulated energy starts to discharge. Current *i*_L_ is now determined by the sum of capacitor current *i*_C13_ and current *i*_p_. The explanation refers to Figure 15, Figure 16 and Figure 17 and Figure 19.

#### 3.3.3. Time Interval *t*_2_ ≤ *t* < *t*_3_

**Figure 20:**
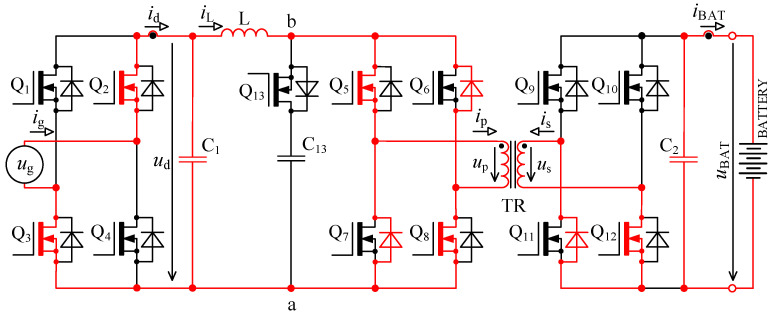
Discharging mode–time interval *t*_2_ ≤ *t* < *t*_3_.

In time instant *t*_2_, transistors Q_9_ and Q_13_ are turned off. The secondary current *i*_s_ must maintain its direction and flows through the body diode of transistor Q_11_. At the same time, the transistor Q_13_ current is interrupted. Next, the current *i*_p_ drops. Transformer leakage inductance tries to maintain the current through the transformer *i*_p_ magnitude. This causes a decrease in *u*_p_ voltage. The explanation refers to Figure 15, Figure 16 and Figure 17 and Figure 20.

#### 3.3.4. Time Interval *t*_3_ ≤ *t* < *t*_4_

**Figure 21:**
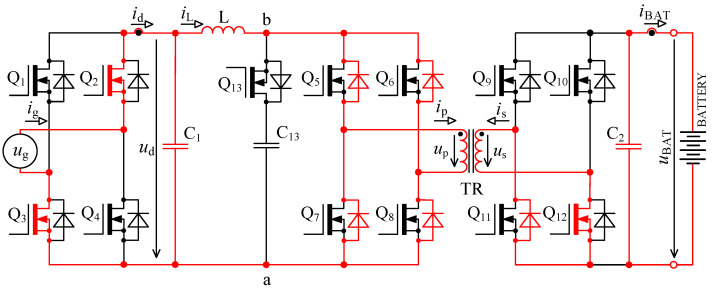
Discharging mode–time interval *t*_3_; discharging mode–time interval *t*_4_ ≤ *t* < ½*T*. *t* < *t*_4_.

In time instant *t*_3_ transistors Q_5_ and Q_8_ are turned off. Inductor current *i*_L_ maintains its direction even after the end of energy transfer from the transformer. Current *i*_L_ flows back through the body diodes of transistors Q_5_ to Q_8_. This keeps the primary winding of transformer TR voltage close to zero. The explanation refers to Figure 15, Figure 16 and Figure 17 and Figure 21.

#### 3.3.5. Time Interval *t*_4_ ≤ *t* < ½*T*

**Figure 22:**
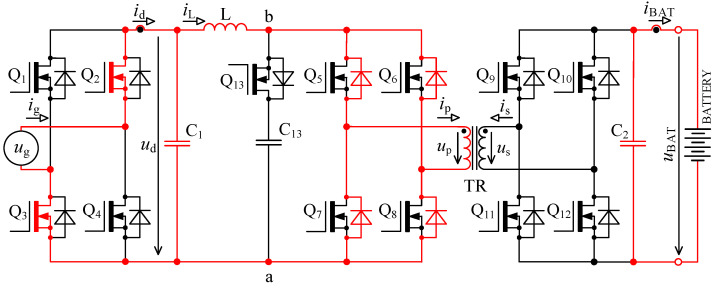
Discharging mode–time interval *t_4_* ≤ *t < ½T*.

In time instant *t*_4_, the energy accumulated in the leakage inductance of the secondary winding is depleted and, therefore, the transistor Q_12_ is turned off. Other transistors were already turned off in the previous time interval. This allows zero current switching on during the next time interval. The explanation refers to Figure 15, Figure 16 and Figure 17 and Figure 22.

#### 3.3.6. Time Interval ½*T* ≤ *t* < (*t*_1_ + ½*T*)

In time instant ½*T* transistors Q_10_, Q_11_ are turned on, and transistors Q_5_ to Q_8_, Q_10_, Q_11_ remain turned on. Current *i*_p_ flows back through the body diodes of transistors Q_6_ and Q_7_. If the voltage *u*_p_ exceeds the magnitude of capacitor C_13_ voltage, the current through the body diode of transistor Q_13_ starts to charge the capacitor C_13_. Waveforms of voltages and currents are analogous to what was described in Section 3.3.1.

#### 3.3.7. Time Interval (*t*_1_ + ½*T*) ≤ *t* < (*t*_2_ + ½*T*)

In time instant (*t*_1_ + ½*T*), transistors Q_6_, Q_7_ and Q_13_ are turned on, and energy from the battery is transferred into power grid. Waveforms of voltages and currents are analogous to what was described in Section 3.3.2.

#### 3.3.8. Time Interval (*t*_2_ + ½*T*) ≤ *t* < (*t*_3_ + ½*T*)

In time instant *(t*_2_ + ½*T)*, transistors Q_10_ and Q_13_ are turned off. The secondary current *i*_s_ must maintain its direction and flows back through the body diode of transistor Q_12_. At the same time, the transistor Q_13_ current is interrupted. Waveforms of voltages and currents are analogous to what was described in Section 3.3.3.

#### 3.3.9. Time Interval (*t*_3_ + ½*T*) ≤ *t* < (*t*_4_ + ½*T*)

In time instant *(t*_3_ *+* ½*T)*, transistors Q_6_ and Q_7_ are turned off. Current *i*_L_ flows back through the body diodes of transistors Q_5_ to Q_8_. Waveforms of voltages and currents are analogous to what was described in Section 3.3.4.

#### 3.3.10. Time Interval (*t*_4_ + ½*T*) ≤ *t* < *T*

In time instant *(t*_4_ *+* ½*T)*, transistor Q_11_ is turned off. Other transistors were turned off during the previous time interval. Waveforms of voltages and currents are analogous to what was described in Section 3.3.5.

## 4. New Prototype of a Bidirectional Charger Enhanced with Active Element “D”

**Figure 23:**
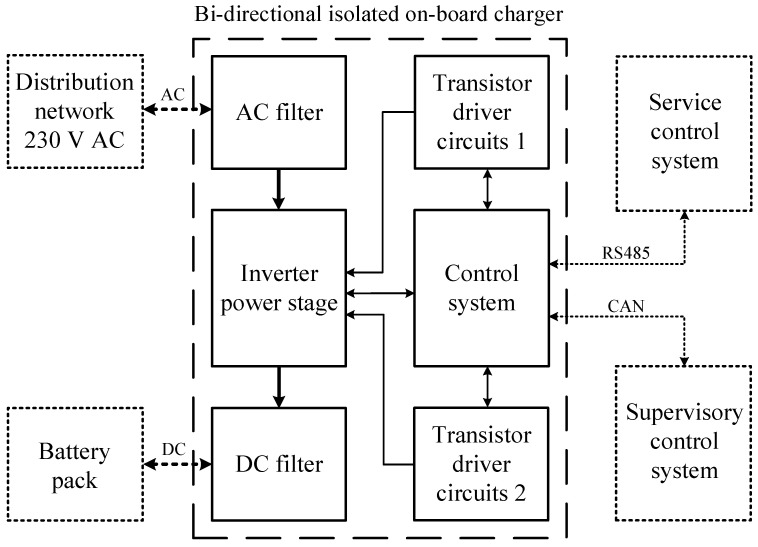
Block diagram of main modules of bidirectional isolated on-board charger. Each block represents one printed circuit board.

To verify the modified topology of the bidirectional charger, a new prototype enhanced with an active element “D” was assembled. The new prototype consists of six interconnected PCBs: AC filter, inverter power stage, DC filter, control system, transistor driver circuits 1 and transistor driver circuit 2, as shown in Figure 23. The main parameters of the bidirectional charger are shown in Table 1. 

Inverter power stage contains four power transistors Q_1_ to Q_4_ type IPW60R017C7XKSA1, see bridge “A” in Figure 5 and five power transistors Q_5_ to Q_8_ and Q_13_ type C3M0032120K, see bridge “B” and active element “D”, four power transistors Q_9_ to Q_12_ type C3M0030090K, see bridge “C” in Figure 5.

For *i*_d_ and *i*_BAT_ measurements, galvanically isolated current sensors ACS730-KLCTR-50AB-T are used, for *u*_g_, *u*_d_ and *u*_BAT_ measurements, precision optically isolated voltage sensors ACPL-C87A-500E are used. Capacitors C_1_, C_2_ and C_13_ are able to provide high peak current and are durable and as small as possible. Therefore, they were assembled as a combination of ceramic and foil capacitors. 

C_1_: 7 × 0.33 μF/630 V foil + 7 × 100 nF/1.5 kV X7R ceramic.

C_2_: 11 × 1 μF/630 V foil + 11 × 100 nF/1.5 kV X7R ceramic.

C_13_: 4 × 33 nF/1kV + 6 × 22 nF/1kV special pulse ceramic. 

Transistor driver circuit 1 contains six drivers of ADUM4135BRWZ type for transistors Q_3_, Q_4_, Q_6_, Q_8_, Q_10_ and Q_12_ and five pieces of galvanically isolated DC/DC power supplies. 

Transistor driver circuit 2 contains seven drivers of ADUM4135BRWZ type for transistors Q_1_, Q_2_, Q_5_, Q_7_, Q_9_, Q_11_ and Q_13_ and seven pieces of galvanically isolated DC/DC power supplies. 

Control system contains five galvanically isolated power supplies, linear regulators (5, 3.3 V), 3.3 V voltage reference for AD converter and analogue supply for microcontroller, circuit for isolated measurement of voltages and currents, two high-speed CAN transceivers, service communication interface with RS485, temperature measurement and 32-bit microcontroller ARM Cortex M4, STM32F446XC (STMicroelectronics, Geneva, Switzerland).

Microcontroller STM32F446XC works at 180 MHz system clock, and five timers are used to control charging and discharging mode. For every single transistor control, specific timer output was assigned. For example, channel 1 of timer TIM_1_ for transistor pair Q_5_, Q_8_. All timers utilise asymmetric PWM. To better understand the control algorithms, logic signals for charging mode are shown in Figure 9 and in Figure 15 for discharging mode. Duration labelled as *t*_delay_ is in charging mode realised with phase shift of timers. In discharging mode the duration *t*_delay_ for transistors Q_5_ to Q_8_ is realised with timer dead time and for transistor Q_13_ with phase shift of timer.

Considering the requirements for as small as possible dimensions and weight, fluid cooling was selected for transistor cooling. The simulation focused on thermal transfer, and fluid movement in the heatsink was performed in software COMSOL Multiphysics 5.5 (COMSOL Inc., Stockholm, Sweden). For both charging and discharging modes, the temperature 80 °C without radiation effects was assumed. All the heat is dissipated through the heatsink. This simplification corresponds to real conditions in the charger, whose internal space is heated-up with power dissipation of the remaining components. To prevent overheating damage of power transistors, simulations of the maximal possible temperature of cooling fluid were performed. It showed that it is possible to reach up to 110 °C of cooling fluid without exceeding the absolute maximum temperature rating of the transistor junction.

Two variants of transistor Q_1_ to Q_13_ control techniques were simulated in charging and discharging modes.

In the first variant, referred to as “Uncontrolled”, only transistors Q_5_ to Q_8_ and Q_13_ were driven in charging mode and only transistors Q_1_ to Q_4_, Q_9_ to Q_12_ and Q_13_ were driven in discharging mode. In the second variant, referred to as “Controlled”, all transistors were actively driven.

The inlet temperature of the coolant is 80 °C at a flow rate of 2 L/min.

**Figure 24:**
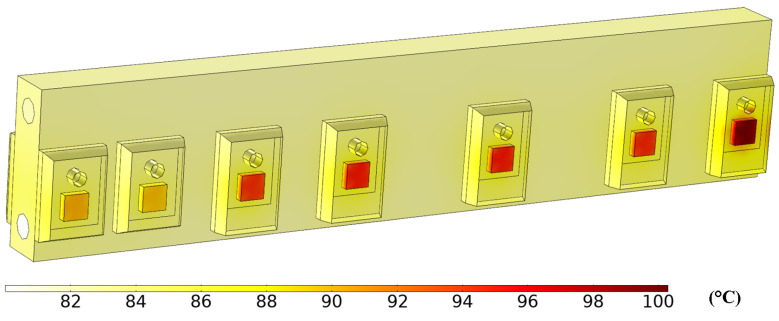
Transistor temperatures in charging mode and active control of all transistors. Left to right: Q_1_, Q_2_, Q_13_, Q_5_, Q_7_, Q_11_ a Q_9_.

Figure 24 shows the example of temperature simulation of all transistors placed on the heatsink. This is for maximal power of the charger and active control of all transistors in charging mode. Coolant inlet temperature was 80 °C, and outlet temperature was 82.2 °C at a flow rate of 2 L/min.

**Figure 25:**
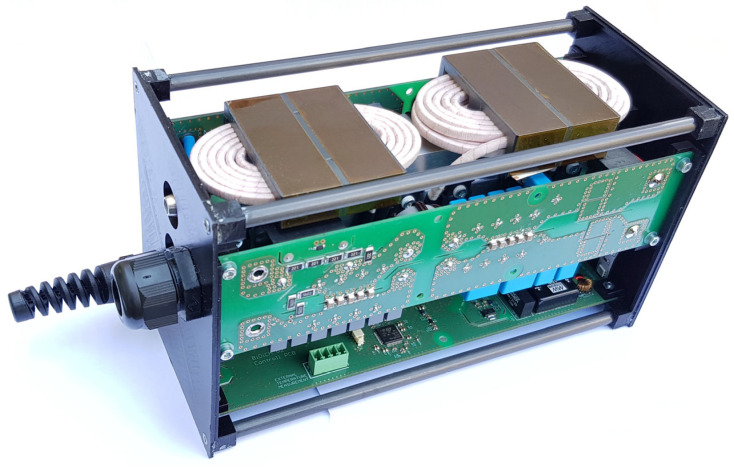
A photograph of the second prototype of the bidirectional isolated on-board charger.

Figure 25 shows a photograph of the new prototype of the bidirectional charger enhanced with an active element “D”. Dimensions: 224 × 100 × 130 mm (W × D × H).

## 5. The Experimental Microgrid Platform

**Figure 26:**
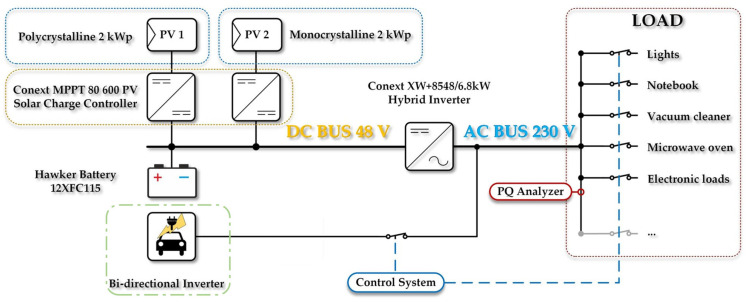
Diagram of the entire test platform.

The experimental microgrid platform for the development of the Vehicle to Grid technologies simulates the electricity consumption of a typical single-family home. The microgrid system is based on a hybrid AC/DC architecture. The centre of the experimental platform is a Conext XW+ 8548 E hybrid inverter (Schneider Electric SE, Rueil-Malmaison, France). The testing platform consists of two parts. The first part is based on the main 48 V DC bus, whereas the second is based on the 230 V AC bus. The DC part consists of two Conext 80 600 DC/DC-MPPT PV inverters (Schneider Electric SE, Rueil-Malmaison, France). These PV inverters convert electricity to supply appliances and storage batteries from photovoltaic strings. The batteries are thin plate pure lead Hawker 12XFC115. One battery’s nominal voltage and capacity are 12 V and 115 Ah. These batteries are connected in a battery bank, forming four groups, each including four batteries. The 48 V DC bus voltage varies from 40.5 to 64 V DC depending on the battery’s state of charge and the charging process. The rated power of each of the two PV strings is 2 kW. The second part of the platform is based on a 230 V AC bus with a frequency of 50 Hz. The AC bus is drawn directly from the hybrid inverter to which the individual household appliances are connected. Figure 26 shows a schematic of the microgrid system used to perform the experiments in this article.

Every part of testing platform communicates between self. The authors’ previous work in the field of power-line communication (PLC) technology describes potential concepts of the real-time communication in off-grid electrical systems [40].

## 6. Results and Discussion

For experimental verification of the bidirectional charger, a new prototype enhanced with an active element “D” was assembled, and a new control strategy of transistors Q_9_ to Q_12_ was used. The measurement station mainly consisted of the following instruments: Rigol MSO4054 oscilloscope, Micsig DP 10013 high voltage differential probe, Rigol RP1002C current probe, PEM CWTUM/03/B ultra-miniature HF current probe.

**Figure 27:**
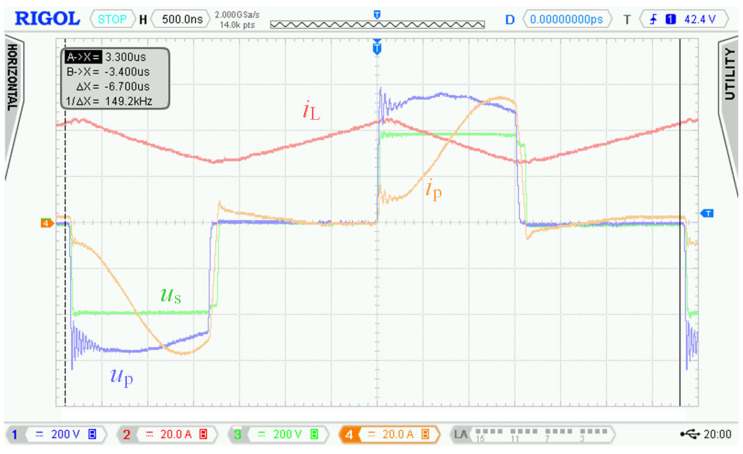
Experimental measurement in charging mode with active element “D”. Measured at: *u*_g_ = 240.3 V, *i*_g_ = 33.5 A, *p*_g_ = 8049.9 W, *u*_BAT_ = 371.1 V, *i*_BAT_ = 20.5 A, *f*_s_ = 150 kHz. Channel description: 1 = *u*_p_, 2 = *i*_L_, 3 = *u*_s_, 4 = *i*_p_.

In Section 2.2, waveforms of voltages and currents in charging mode with inductor inductance 25 μH were shown. Those waveforms were acquired while using the new topology according to Figure 1, i.e., without active element “D”. Figure 27 shows waveforms of voltages and currents in charging mode with the topology according to Figure 5, i.e., with the active element “D”. Comparing Figure 4 and Figure 27, it is obvious that *u*_p_ and *u*_ab_ overvoltage problem, caused by transformer leakage inductance, was solved by the active element “D”. Voltage *u*_p_ waveform after modification contains higher frequency oscillations caused by other parasitic properties, which were not present with the topology according to Figure 1. The root cause of these oscillations is mainly the parasitic properties of PCBs and electronic components. These oscillations have much lower amplitude and do not overstress the semiconductors with overvoltage. Voltage *u*_p_ amplitude is, in this case, equal to 600 V and did not exceed the absolute maximum voltage of 700 V, see Section 3.1. Dumped resonances of voltage *u*_s_ (Figure 4), caused by circuit parasitic on the secondary side of the transformer, were successfully suppressed with alternative control strategy of transistors Q_9_ to Q_12_, different to what was mentioned in Section 2.1.

### 6.1. Experimental Measurement Results Acquired in Charging Mode 

Figure 27 shows waveforms of the different quantities during single switching period. Measurement was taken at nominal power and represented its specific time instant. 

**Figure 28:**
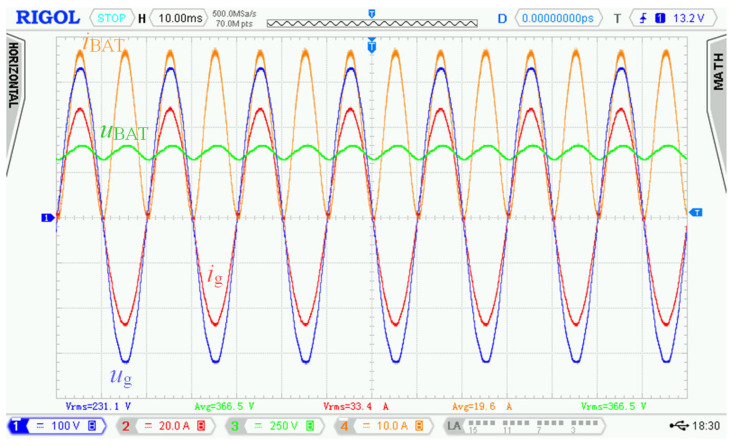
Experimental waveforms of voltages and currents measured in charging mode of bidirectional charger connected to 230 V AC power grid. Requested battery current *I*_BATr_ = 20 A. Measured values: *U*_g_ = 231.1 V, *I*_g_ = 33.4 A, *U*_BAT_ = 366.5 V, *I*_BAT_ = 19.6 A. Channel description: 1 = *u*_g_, 2 = *i*_g_, 3 = *u*_BAT_, 4 = *i*_BAT_.

Figure 28 shows the voltage and current waveforms over a time interval of several periods of the 50 Hz power grid at rated power. The figure implies that waveforms of power grid current *i*_g_ follow the shape of power grid voltage *u*_g_ with minimal total harmonic distortions and phase shift (unity power factor). 

Distortion of battery current *i*_BAT_ was caused by dynamic properties of the used electronic artificial load connected to the battery. This electronic load substituted insufficient battery capacity as well as dissipated energy during charging mode. This prevented repetitive battery cycling during the experimental measurements.

The control principle supports very high dynamics of change of power grid current *i*_g_, but its dynamics is reduced with respect to requirements and usage of the bidirectional charger.

### 6.2. Experimental Measurement Results Acquired in Discharging Mode 

**Figure 29:**
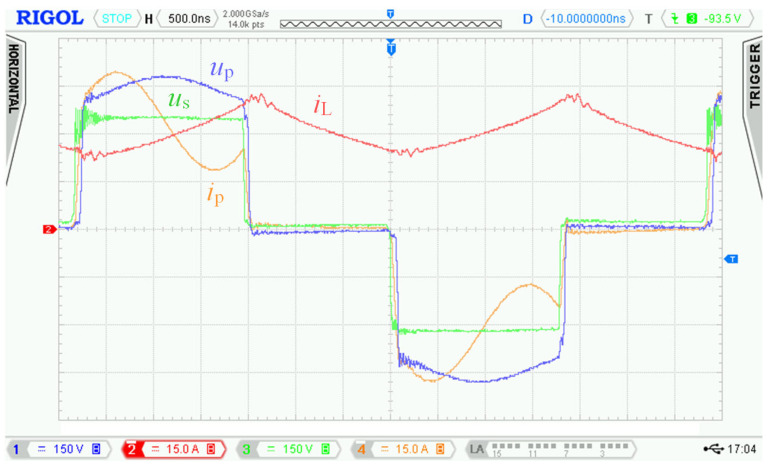
Experimental measurement in discharging mode with active element “D”. Measured at: *t*_on_ = 1.79 μs, *t*_Q13_ = 1.71 μs, *t*_delay_ = 0.08 μs, *u*_g_ = 229.1 V, *i*_g_ = 31.2 A, *p*_g_ = 7147.9 W, *u*_BAT_ = 330.4 V, *i*_BAT_ = 22.6 A, *f*_s_ = 150 kHz. Channel description: 1 = *u*_p_, 2 = *i*_L_, 3 = *u*_s_, 4 = *i*_p_.

Figure 29 shows the voltage and current waveforms during one single switching period. It shows the values corresponding to a specific time interval at rated power. 

**Figure 30:**
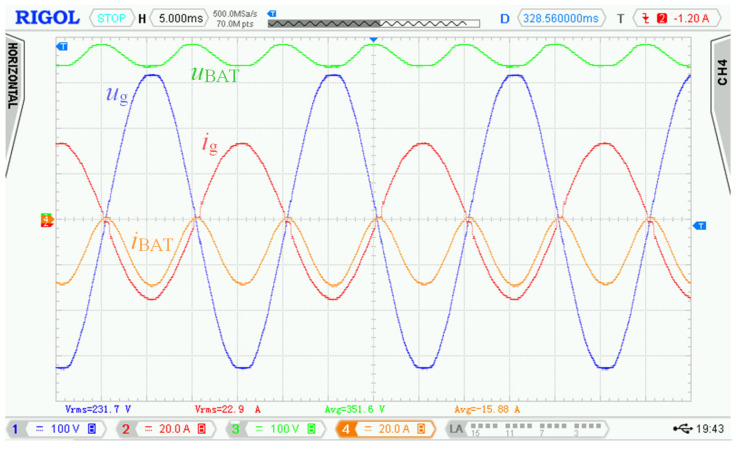
Experimental waveforms of voltages and currents in discharging mode and bidirectional charger connected to power grid 230 V AC. Requested battery current *I*_BATr_ = 15 A (3 periods of power grid). Measured values: *U*_g_ = 231.7 V, *I*_g_ = 22.9 A, *U*_BAT_ = 351.6 V, *I*_BAT_ = 15.9 A. Channel description: 1 = *u*_g_, 2 = *i*_g_, 3 = *u*_BAT_, 4 = *i*_BAT_.

Figure 30 shows the voltage and current waveforms over a time interval of three periods of the 50 Hz power grid. The waveforms also indicate minimal harmonic distortion, similarly to the charging mode. 

Optimal function and maximum efficiency in charging and discharging mode is achieved with active control of all transistors Q_1_ to Q_13_.

**Figure 31:**
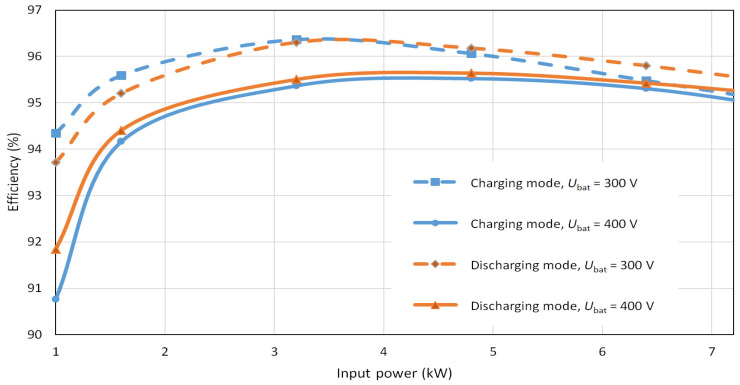
Measured efficiency of the bidirectional galvanically isolated on-board charger.

Figure 31 shows the efficiency curve of the bidirectional charger for battery voltages of 300 and 400 V. The maximum efficiency of the bidirectional charger in charging mode for a battery voltage of 300 V was 96.4% at 3.4 kW and for a battery voltage of 400 V was 95.6% at 4.6 kW. The maximum efficiency in discharge mode for 300 V battery was 96.3% at 3.8 kW and for 400 V battery, voltage was 95.7% at 4.4 kW, see Figure 31.

The following EMC measurements were carried out on the new prototype of the bidirectional charger:

Conducted Emissions Tests (CE)

- CE02—Conducted Emission, Voltage method (IEC CISPR 25)

Radiated Emissions Tests (RE)

- RE01, Radiated Emission, ALSE method (IEC CISPR 25)

Immunity to ElectroStatic Discharges Tests (ESD)

- ESD02, Unpowered test (ISO 10605)
- TP01, Immunity to transient pulse 1 and 1b (ISO 7637-2)
- TP02, Immunity to transient pulse 2 (ISO 7637-2)
- TP03, Immunity to transient pulse 3a and 3b (ISO 7637-2)
- TP04, Immunity to transient pulse 4 (ISO 7637-2)
- TP05, Immunity to transient pulse 5 (ISO 7637-2)

The results of all these EMC measurements meet the given criteria.

**Figure 32:**
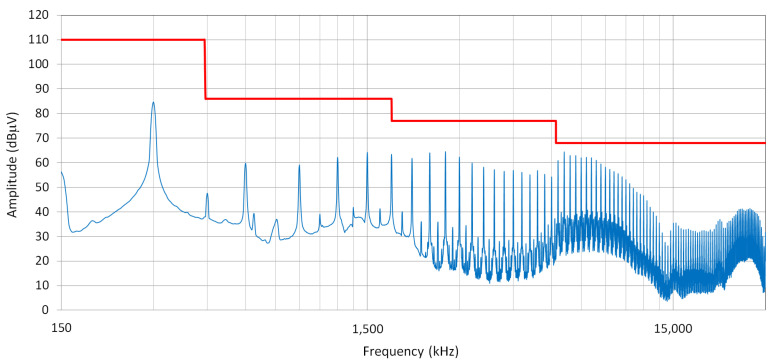
Conducted emissions testing. Voltage method according to CISPR 25. *U*_g_ = 230 V, *I*_g_ = 15 A.

Figure 32 shows the emissions testing waveform for the bidirectional charger.

## 7. Conclusions

This paper describes the research, development and experimental testing of a bidirectional galvanically isolated on-board charger for electric vehicles. The main objective was to meet the requirements of 4 kW/kg and 2 kW/dm^3^, bidirectional power control and galvanic insulation from the power grid. The power density of the realised charger was 4 kW/kg and 2.46 kW/dm^3^, and the maximum efficiency was 96.4% at 3.4 kW. To be able to achieve such low weight and values, specific electronic and mechanical elements had to be addressed. As for the electronic circuits, we mostly addressed the inductor and transformer, and as for the mechanical components, the solution concerned the heatsink. To achieve as low weight and volume as possible, fluid cooling was used to cool down the power transistors. To make the inductor and transformer smaller, high switching frequency of 150 kHz had to be used. 

Based on the state-of-the-art research, a circuit scheme of the bidirectional charger was proposed, and a prototype for testing the control algorithms was built. In fact, several prototypes were built, two most prominent being described in this paper. The first prototype of the charger is described in Section 2, where problems and their solutions are also dealt with. Due to the parasitic properties of the transformer and other electronic circuits, overvoltage and oscillations occurred across the primary winding of the transformer. There was also dumped resonance across the secondary winding. Those undesirable effects were eliminated with topology modification by adding an active element “D” into the charger and with new Q_9_ to Q_13_ control strategy. The feasibility of this solution was verified with a new prototype of the bidirectional charger, which is described in Section 4. The novel switching strategy of Q_9_ to Q_13_ transistors’ control and modified topology with an active element “D” were patented. The realised charger has an output of either 7.2 kW if it uses only single-phase or 21.6 kW if it uses three-phases.

The contribution of this work can be summarised as follows:

- The single-stage inverter topology was modified to eliminate undesirable phenomena.
- Bidirectional power control and galvanic insulation from the power grid.
- Owing to the novel switching strategy of Q_9_ to Q_12_ transistors’ control, the charger does not need RCD snubbers in order to limit the voltage spikes caused by leakage inductance of the transformer. This has the effect of reducing losses, increasing efficiency and also reducing weight and volume.
- The proposed transistor switching control method eliminates unwanted HF oscillations on both the primary and secondary sides of the transformer in the on-board charger.
- In order to reduce the size and weight of the inductor and transformer, high switching frequency of 150 kHz was used.
- The charger allows V2G technology.
- The power density of the new charger was 4 kW/kg and 2.46 kW/dm^3^, and the maximum efficiency was 96.4% at 3.4 kW.
- The new charger has an output of either 7.2 kW if it uses only single-phase, or 21.6 kW if it uses three-phases.

## 8. Patents

The solution presented in the paper has been patented. The name of the patent is “Charger for bidirectional energy flow and controlling it”. The patent number is PUV 2020-37617 (308969), CZ2020236A3. 

## Figures and Tables

**Table 1:** Major parameters of the bidirectional on-board charger.

Parameter	Value
Grid AC Voltage	230 V + 10%
Battery DC Voltage	300 to 420 V
Maximum grid current	32 A RMS
Maximum battery current	20 A
Nominal input power	7.2 kW
AC grid frequency	50 Hz
Switching frequency	150 kHz
Inductance L	25 μH
Capacity C_1_	3.01 μF
Capacity C_2_	12.1 μF
Capacity C_13_	270 nF
Transformer windings	4 primary turns, 3 secondary turns
Weight	1.8 kg
Volume	2.92 dm^3^
Q_1_–Q_4_, CoolMOS PowerTransistor IPW60R017C7XKSA1	(600 V, 129 A, R_DS(on)_ = 17 mΩ, *U*_SD_ = 0.9 V ^1^ @ *I*_F_ = 58.2 A)
Q_5_–Q_8_, Q_13_, Silicon Carbide Power MOSFET C3M0032120K	(1200 V, 63 A, R_DS(on)_ = 32 mΩ, *U*_SD_ = 4.6 V ^1^ @ *I*_F_ = 20 A)
Q_9_–Q_12_, Silicon Carbide Power MOSFET C3M0030090K	(900 V, 63 A, R_DS(on)_ = 30 mΩ, *U*_SD_ = 4.8 V ^1^ @ *I*_F_ = 17.5 A)

^1^*U*_SD_ is body diode forward voltage.

## Data Availability

Not applicable.

## Patent Annotation

Charger for bidirectional energy flow, which has on the way from the mains voltage source (*u*_g_) to the battery: the first bidirectional converter (A) consisting of switches (S_1_ to S_4_) operating on the mains frequency, the second bidirectional converter (B) consisting of switches (S_5_ to S_8_) operating at a frequency in the order of hundreds of kHz, a transformer (TR) and a third bidirectional converter (C) consisting of switches (S_9_ to S_12_) operating at the same frequency as the second converter. A coil (L) is connected in one of the buses (+, −) connecting the first and second converters (B, C), behind which the buses (+, −) are connected via a separate switch (S_13_) and a capacitor (C_13_). The method of controlling the charger by the control unit for the mains battery charging phase consists of a signal from the control unit being switched by a switch (S_13_) with period T/2 and the second and third converters (B, C) are switched with period *T*. For safety reasons, the control unit leaves the switch (S_13_) open for additional time during the safety delays *t*_dead_ and *t*_delay_ before the interval *t*_0_, or after this interval *t*_0_.
